# Mapping the Literature on Diet and Multiple Sclerosis: A Data-Driven Approach

**DOI:** 10.3390/nu14224820

**Published:** 2022-11-14

**Authors:** Xiaochen Qu, Erin I. Walsh, Nicolas Cherbuin, Lucinda J. Black

**Affiliations:** 1 Curtin School of Population Health, Curtin University, Bentley, WA 6102, Australia; 2 Population Health Exchange, National Centre for Epidemiology and Population Health, Australian National University, Canberra, ACT 2615, Australia; 3 Department of Health Economics Wellbeing and Society, National Centre for Epidemiology and Population Health, Australian National University, Canberra, ACT 2615, Australia; 4 Curtin Health Innovation Research Institute (CHIRI), Curtin University, Bentley, WA 6102, Australia

**Keywords:** diet, nutrition, multiple sclerosis, citation network analysis, text mining

## Abstract

Multiple sclerosis (MS) is a chronic neurological disease of the central nervous system that is currently incurable. Diet may influence the onset and progression of MS. A variety of literature reviews have been conducted in the field of diet and MS. However, conventional reviews mostly focus on specific topics rather than delivering a holistic view of the literature landscape. Using a data-driven approach, we aimed to provide an overview of the literature on diet and MS, revealing gaps in knowledge. We conducted citation network analysis to identify clusters of all available publications about diet and MS over the past 50 years. We also conducted topic analysis of each cluster and illustrated them in word clouds. Four main clusters were identified from 1626 publications: MS risk and symptom management; mouse models of MS; gluten sensitivity; and dysphagia. Citation network analysis revealed that in this emerging field, articles published after 1991 were more likely to be highly cited. Relatively few studies focused on MS disease progression compared to risk factors, and limited evidence was available for many foods and nutrients in relation to MS. Future studies could focus on filling these identified knowledge gaps.

## 1. Introduction

Multiple sclerosis (MS) is a chronic neurological disease of the central nervous system (CNS) that affects more than two million people worldwide [1]. The aetiology is multifactorial, and both genetic and environmental risk factors have been demonstrated to play a role [1]. The symptoms may include numbness, muscle weakness and tremors, motor and vision impairment, cognitive decline, as well as bowel and bladder disturbances [2]. The onset of MS is typically between 20 and 40 years of age [3]. Although there is no known cure, diet is a potential factor that may influence the disease onset and progression [4,5,6]. The literature on diet and MS covers a broad range of topics, such as therapeutic diets, certain foods and nutrients, disease onset and progression. There are numerous scoping and systematic literature reviews summarising and synthesising information from the literature on diet and MS [7,8,9]. Such reviews are useful in helping readers understand specific topics related to diet and MS research. However, they are limited to relatively narrow research questions in order to explore and meaningfully summarise the findings. Therefore, such reviews tend to be highly specialised [10]. Literature reviews on diet and MS may focus on specific nutrients, such as vitamin D or polyunsaturated fatty acids (PUFAs) and MS onset or progression [11], mechanisms for dietary factors influencing MS disease outcomes from the pathophysiology perspective [4,9], or dietary interventions for MS [12,13]. Among studies on diet and MS, certain foods and nutrients are more frequently investigated than others. For example, vitamin D status and PUFAs are widely studied, while the literature on other nutrients is limited. An implication of such focused interests is that they may obscure gaps in knowledge or fail to identify related research or synergistic findings that have been published in different fields.

In contrast to literature reviews, the wide-ranging nature of literature mapping lends itself to providing an overview of the broad field of research [10]. Literature mapping using a data-driven approach relies less on the manual selection of articles and more on automated methods. When mapping the literature on diet and MS, studies about other neurodegenerative diseases, such as Alzheimer’s disease, can be included if there are citation relationships. Many factors, such as oxidative stress, neuroinflammation and apoptosis may cause neurodegeneration [14]. Therefore, foods or nutrients affecting neurodegeneration may be similar across neurodegenerative diseases.

Literature mapping can help researchers and other professionals in the field gain a holistic view of research on diet and MS, including which specific diets or nutrients have been intensively researched in relation to MS, and which need to be studied further. To our knowledge, literature mapping has not been conducted for the field of diet and MS. Hence, we aimed to map the existing literature relating to diet and MS using four steps, as previously developed by our team [10]: (i) defining the search terms and searching for the literature systematically; (ii) recording the full title of the articles, as well as the citation links; (iii) identifying clusters using citation network analysis; and (iv) characterising clusters by extracting topics from article titles using text mining techniques [10]. The goal was to provide a landscape of the literature on diet and MS, helping to identify trends and gaps, and guiding future research questions.

## 2. Materials and Methods

### 2.1. Literature Search

The literature search was carried out on 9th December 2021 on Web of Science Core Collection Database. Search terms ((diet OR nutri* OR aliment*) AND ((“multiple sclerosis”) OR (demyelin*) OR (clinically isolated syndrome))) were used to search in “topics”, which includes titles of articles, abstracts, author keywords, and “KeyWords Plus”. Only published articles and review articles were included; other publications, such as editorial materials, book chapters, and articles with early access were excluded. Articles were exported with full records including information including authors, titles, source, year of publication and Digital Object Identifier (DOI).

### 2.2. Citation Network Analysis and Clustering

All documents exported from Web of Science Core Collection Database were imported to CitNetExplorer for citation network analysis and cluster identification [15]. In CitNetExplorer, articles are allocated to different clusters by calculating the quality function, which is higher when more articles have a direct citation relationship with each other [16]. When the function is maximised, the articles are allocated into one cluster. As a result, one publication is assigned to one cluster or another, or not assigned to any cluster [16]. Some articles may not be included in a cluster although they relate to the same topic, if they are not sufficiently related (with direct citation) to the articles in the cluster. Typically, a minimum cluster size is set (e.g., 20 articles in our study) to obtain valid and interpretable results. If this threshold is not met, citations are omitted. After a first-level clustering, a “drill down” function is used to segment each cluster into sub-clusters. This process is repeated iteratively on sub-clusters until they cannot be divided further, or the number of publications in the cluster is less than 20. Further, visualisation of citation networks is used to facilitate the analysis of clusters.

### 2.3. Cluster Characteristics

Publications were exported from CitNetExplorer by clusters and sub-clusters for text mining and cluster characterisation with R [17]. We converted titles of publications into a corpus (collection of natural language documents) using the “tm” package (version 0.7-8, Feinerer I., Hornik K., Meyer D.). Compound words and their variations were concatenated into one word (e.g., “multiple sclerosis” was collapsed into multscler). Capital letters were then converted to lower case letters, and all punctuation and stop words were removed following text mining conventions [10]. Stop words are words frequently appearing in the corpus, but provide little information (e.g., the, for, in, why, use). Words were stemmed using Porter’s stemming algorithm in the “SnowballC” package (version 0.7.0, Milan Bouchet-Valat) (e.g., “autoimmune” and “autoimmunity” were converted to “autoimmun”). Resultant corpora (the collection of texts) were saved as spreadsheets and converted to Term Document Matrix (TDM) for comparison clouds in the “wordcloud” package (version 2.6, Ian Fellows). Further text mining was conducted to extract topics of clusters using Latent Dirichlet allocation (LDA) in the “topicmodels” package (version 0.2-12, Grün B., Hornik K.).

Word clouds were generated for each cluster and sub-cluster, allowing the most frequently investigated topics and keywords from a large amount of literature, to be easily observed. Further, comparison clouds were plotted to show the unique topic words in each cluster. The comparison cloud compared the frequencies of words across sub-clusters in each cluster. When a word appeared only in one sub-cluster frequently, but was rarely seen in other sub-clusters, it would be depicted with a bigger font in the comparison cloud.

Text mining was conducted using LDA, which extracted topics from titles of articles from each cluster. LDA is a generative statistical model based on Bayes’ Theorem, which makes it possible to infer probabilities of unobserved value (the topic of an article) from the observations (titles of articles). Each term in documents (here corpora of article titles) has possible underlying topics in LDA, and each topic is a mixture of words. The topic probabilities reflect how much they can represent the document.

## 3. Results

**Figure 1:**
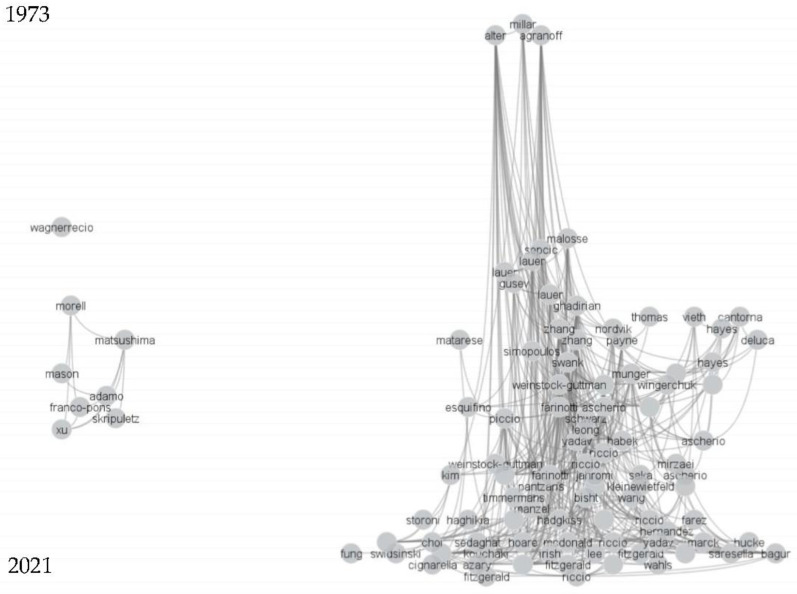
Citation networks of 100 most cited articles among all articles exported from Web of Science Core Collection.

In total, 1626 eligible articles were exported from the Web of Science Core Collection Database and imported into CitNetExplorer for citation network analysis (Supplementary File S1). The 100 most cited articles are shown in Figure 1. The earliest article found on diet and MS in the database was published in 1973. None of the articles in the top 100 most cited were published between 1974 and 1991. From 1991 onwards, articles in this field became more numerous, with more highly cited articles.

### 3.1. Literature Clustering

Due to the minimum size requirement of clusters (*n* = 20 articles), 678 articles were not identified as belonging to any cluster according to the algorithm of CitNetExplorer and were thus omitted from topic analysis. In total, 948 articles were assigned into four main clusters, which included 728, 166, 32 and 22 articles, respectively. Articles in cluster 1 covered the whole time period from 1973 to 2021, while articles in cluster 2 and 3 were relatively recent, including articles published between 1996 and 2021. In cluster 4, the earliest article was published in 1999, and the latest in 2021.

### 3.2. Frequent Words within Titles

**Figure 2:**
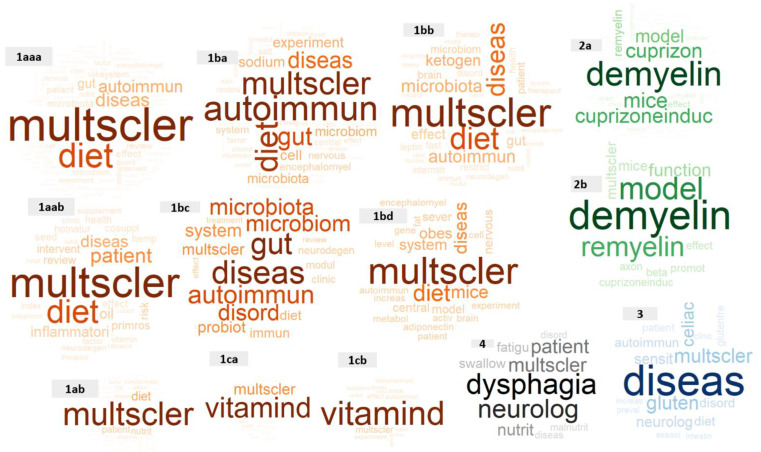
Word clouds of all lowest level clusters and sub-clusters. These lowest level clusters and sub-clusters are those that could not be further divided, including sub-clusters 1aaa, 1aab, 1ab, 1ba, 1bb, 1bc, 1bd, 1ca, 1cb, 2a, 2b, cluster 3 and cluster 4. The larger and more opaque words are those that appeared more frequently in the titles of articles in each sub-cluster. Orange, sub-clusters of cluster 1; green, sub-clusters of cluster 2; blue, cluster 3; and grey, cluster 4. Note: The word size does not represent the frequency of words proportionally across sub-clusters.

Most frequent words reflect predominant concepts for each cluster. Word clouds show the words appearing most frequently in titles of articles belonging to each cluster or sub-cluster (Figure 2; Supplementary Tables S1–S4). Sub-clusters of cluster 1 (orange word clouds) covered a wide range of topics around MS, autoimmune diseases, and diet. While some sub-clusters related to human studies (e.g., “patients”, “people”, “adults”), other sub-clusters were more focused on digestive (e.g., “microbiota”) or cellular processes (e.g., “cell”). Sub-clusters of cluster 2 were regarding demyelination and remyelination with “Cuprizone (mouse) models” being the main study subjects. Cluster 3 had no sub-clusters and focused on celiac disease, gluten, and MS. The most frequent words in cluster 4 were dysphagia and neurology.

### 3.3. Unique Words in Each Cluster

**Figure 3:**
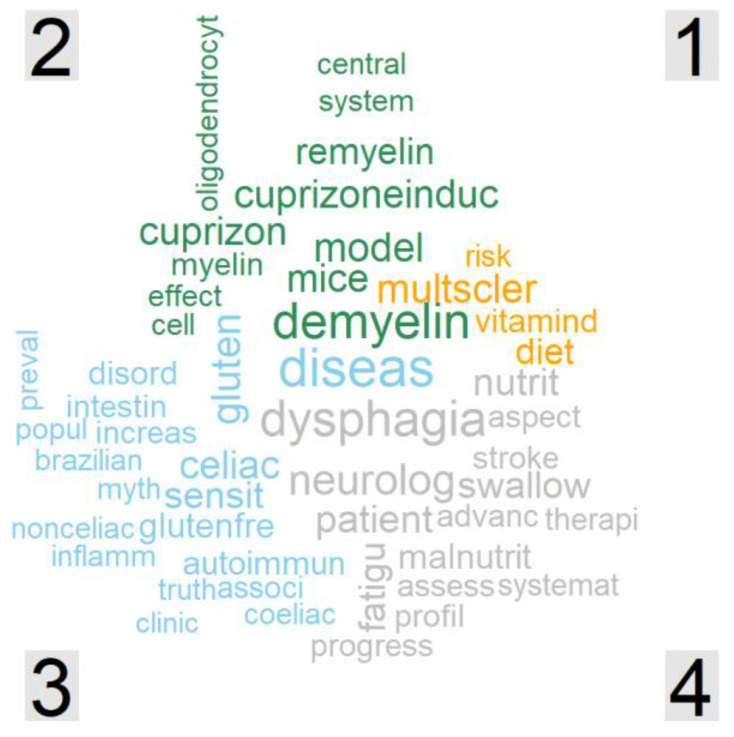
Comparison cloud of cluster 1, 2, 3 and 4. Orange, cluster 1; green, cluster 2; blue, cluster 3; and grey, cluster 4.

Unique words reflect the concepts that best discriminate one cluster from any other. The comparison cloud of cluster 1 to 4 (Figure 3) compared the relative frequency of words and showed the most “unique” words in each cluster or sub-cluster (Supplementary Tables S1–S4).

The most salient unique words of cluster 1 were “multiple sclerosis”, “diet” and “vitamin D”. In cluster 2, they were “demyelination”, “cuprizone” and “(mouse) model”. Cluster 3 was distinguished from other clusters with words such as “(celiac) disease”, “gluten” and “sensitive”. Cluster 4 had the unique words of “dysphagia”, “patient” and “neurology”. Due to the distinctive topics of the four main clusters, the top frequent words in the word clouds of each cluster were largely overlapping with the ones in the comparison cloud.

### 3.4. Topic Analysis

**Figure 4:**
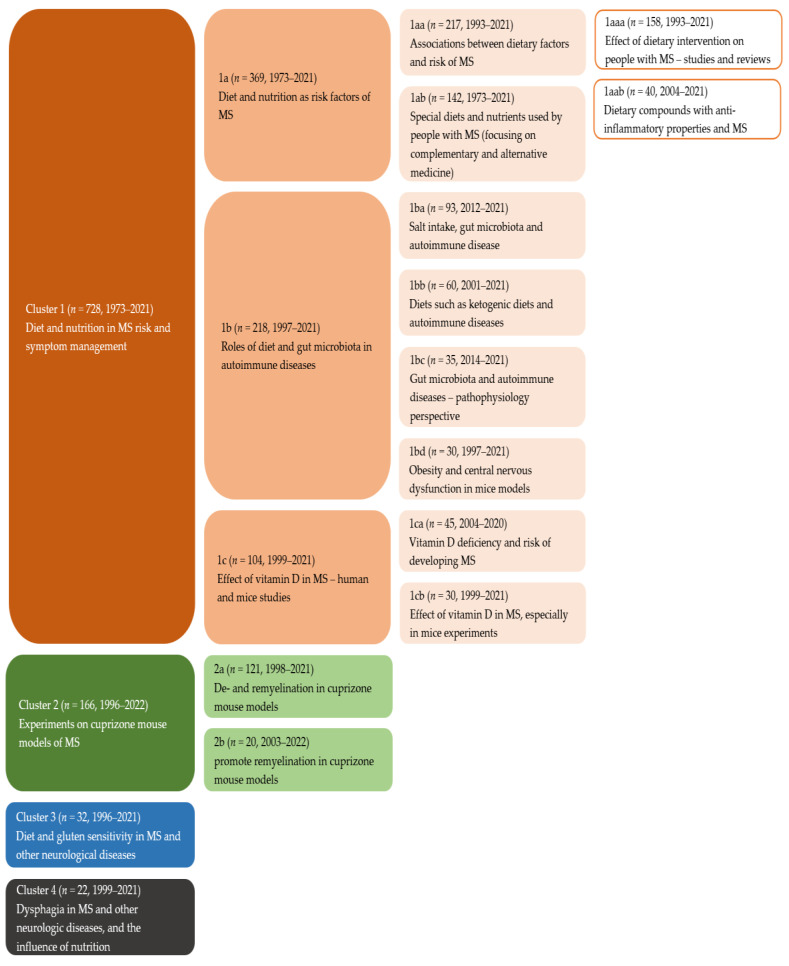
Literature map with topics of clusters and sub-clusters extracted by text mining. Each text box illustrates a cluster or sub-cluster of publications. Alphanumeric characters (1a, 1aa, 1aaa, 1aab, 1ab, 1b, 1ba, 1bb,1bc, 1bd, 1c, 1ca, 1cb, 2a, 2b) have been assigned to clarify the hierarchical relationship between sub-clusters. Orange, cluster 1; green, cluster 2; blue, cluster 3; and grey, cluster 4.

The topic of cluster 1 extracted through text mining could be summarised as diet and nutrition in MS risk and symptom management, with vitamin D as the most highly studied nutrient (Figure 4). Cluster 2 covered studies exploring effects of dietary factors on de- and remyelination in cuprizone mouse models. Articles in cluster 3 reported on diet and gluten sensitivity in MS and other neurological diseases. Cluster 4, with the lowest number of articles, studied dysphagia in MS and other neurologic diseases, and the influence of nutrition.

With three first level sub-clusters, eight second level sub-clusters and two third level sub-clusters, cluster 1 formed the biggest cluster of the literature on diet and MS. In total, 728 articles were included in cluster 1. Sub-cluster 1a, which was its biggest sub-cluster, emphasised diet and risk of MS onset. In this sub-cluster, the diagnosis of MS appeared more frequently as the outcome measure than markers of MS disease progression.

Sub-cluster 1a (*n* = 369) was further divided into two second level sub-clusters, with different topics focusing on associations between dietary factors and risk of MS (sub-cluster 1aa) and special diets, nutrients, and complementary and alternative medicine (sub-cluster 1ab). Within the sub-cluster 1aa, two third-level sub-clusters were detected—intervention studies in people with MS (sub-cluster 1aaa) and studies focusing on anti-inflammatory dietary compounds (sub-cluster 1aab).

Sub-cluster 1b (*n* = 218), which focused on gut microbiota, had four second level sub-clusters. While all investigated gut microbiota, these four sub-clusters approached the topic from different angles: sub-cluster 1ba focused on salt intake using in vitro and in vivo experiments; sub-cluster 1bb researched special diets such as ketogenic diets; sub-cluster 1bc studied the association between gut microbiota and autoimmune diseases such as MS from a pathophysiology perspective; and sub-cluster 1bd included mainly mice studies investigating obesity and central nervous system dysfunction.

Sub-cluster 1c (*n* = 104) focused on studies on vitamin D. Some studies found that vitamin D deficiency was prevalent and had a deleterious impact in inflammatory diseases. Other studies discussed the effects of vitamin D supplementation, safety issues, and factors that may influence circulating 25-hydroxyvitamin D concentrations, such as latitude and ultraviolet (UV) exposure. Studies on both animals and humans were seen in this sub-cluster, as well as review articles.

Cluster 2 was the second biggest cluster and included 166 articles. In contrast to other clusters, which focused on human investigations, articles in cluster 2 were predominantly animal studies, specifically de- and remyelination processes in cuprizone mouse models. Studies in this cluster were divided into two sub-clusters, concerning changes in the body during de- and remyelination (sub-cluster 2a, *n* = 121) [18,19], or the ways to promote remyelination (sub-cluster 2b, *n* = 20) [20]. In this cluster, complex biological changes during de- and remyelination processes were studied in animal models, facilitating the understanding of the mechanisms, and the effect of medicines and supplements [21].

Clusters 3 (*n* = 32) and 4 (*n* = 22) were small compared with clusters 1 and 2. They focused on gluten sensitivity and dysphagia, respectively. Studies in these clusters were not limited to MS but included a broader range of neurological diseases. Studies about dysphagia and MS (cluster 4) only emerged after 1998.

### 3.5. Most Studied Foods and Nutrients

**Figure 5:**
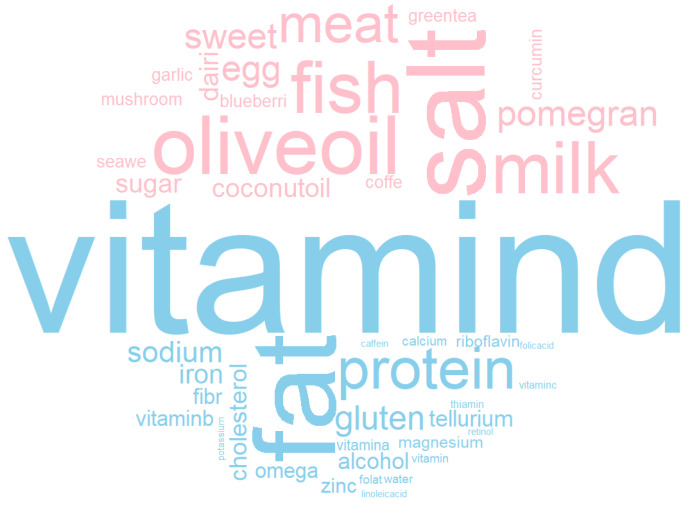
The word cloud of the most investigated foods and nutrients in studies on diet and MS. Words in pink represent most frequently appearing foods, and words in blue represent most frequently appearing nutrients in titles of all articles exported from the Web of Science Core Collection Database. More frequently appeared words are displayed with bigger font sizes.

To explore the most frequently researched foods and nutrients, a word cloud was created (Figure 5). The top food items found in titles of all 1626 articles were salt (11 times), milk and dairy (9 times), olive oil (8 times), fish (7 times), and meat (5 times). Nutrients are more widely investigated than foods. Vitamin D, as the top researched nutrient, was mentioned 131 times in article titles. Fat was the second most studied nutrient, and appeared 118 times, followed by protein (37 times), vitamin B (including folate, folic acid, riboflavin, and thiamine; 22 times), gluten (19 times), sodium (16 times), iron (13 times), cholesterol (12 times), and alcohol (11 times).

### 3.6. Omitted Articles

**Figure 6:**
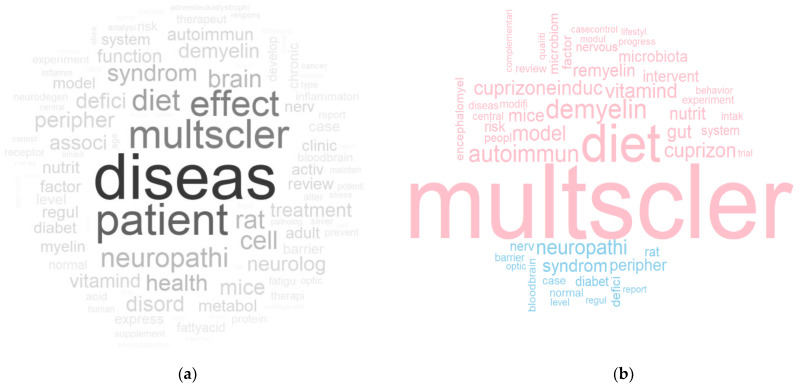
(**a**) The word cloud of omitted articles. More frequently appearing words are displayed with bigger and more opaque fonts. (**b**) The comparison cloud of the four main clusters (pink) and the omitted articles (blue). More frequently appearing words are displayed with bigger font sizes.

In total, 678 articles were omitted during literature clustering due to insufficient citation relationship with other articles. The word cloud showed that the top words in omitted articles covered relatively broad topics about diet and MS (Figure 6a). Words that were frequently mentioned in the four main clusters, such as “patient”, “mice” and “cell”, were also mentioned in omitted articles; however, omitted articles had unique words including “neuropathy”, “peripheral”, “blood–brain barrier”, and “rat” that rarely appeared in the four main clusters (Figure 6b).

In addition, articles mentioning “tellurium” were omitted, including studies investigating the toxicity of tellurium, and neuropathy induced by tellurium using rat models [22,23]. Due to the low number of studies, articles mentioning tellurium did not form a cluster.

## 4. Discussion

Data-driven literature mapping, unlike conventional literature reviews, is not limited to specific topics, but comprehensively considers all available articles in the field. This literature map identifies articles produced over the past five decades in the field of diet and MS, providing a holistic view of the literature landscape. We used a data-driven approach to synthesise 1626 publications into four main clusters which could be characterised as MS risk and symptom management; effects of dietary factors on de- and remyelination in cuprizone mouse models; diet and gluten sensitivity in MS and other neurological diseases; and dysphagia in MS and other neurologic diseases.

Overall, a notable trend was that between 1974 and 1991, few studies investigating diet and MS were published, with no more than two articles found from the Web of Science database every year. After 1991, the links between diet and MS started to be more rigorously studied, and the number of publications has experienced a steady growth, reaching 155 in 2021. Further, an increasing number of foods and nutrients were addressed in the literature since 1991.

Since the aetiology of MS is unclear [24], and there is no known cure, managing the symptoms of MS is the topic of many studies. Making dietary changes is one potential way of improving the symptoms of MS. Nearly half of the retrieved articles were included in cluster 1, which focused on both MS risk and symptom management. While some publications in cluster 1 were published before 1991, most of the sub-clusters only consisted of studies after 1991, such as gut microbiota (sub-cluster 1b), vitamin D (sub-cluster 1c), and anti-inflammatory compounds (sub-cluster 1aab), suggesting that studies on diet and MS risk and symptom management is an emerging field.

Cluster 2 had a strong focus on cuprizone mouse models, which is the most widely used animal model for in vivo experiments on demyelination and remyelination. Articles in this cluster were relatively recent. The earliest article was published in 1996 [25], while the most cited article within this cluster was published in 2001 [26]. The cuprizone mouse model is reproducible and lends itself to the investigation of many aspects of CNS demyelination, such as the gene expression in brains during de- and remyelination, mechanisms of oligodendroglia cells insults, as well as testing the effect of nutrients or foods of interest. Sub-cluster 2b focused on remyelination in cuprizone mouse models, such as dietary factors that might promote remyelination [20,27], and the functional recovery after remyelination [28,29].

The earliest article (1996) in cluster 3 by Hadjivassiliou et al. was highly cited by other articles in this cluster (10 out of 31 articles) and was seminal in identifying gluten sensitivity as a possible contributor to neurological diseases [30]. It stimulated research in this area which specifically investigated the possible benefits of a gluten-free diet and demyelinating diseases such as MS [31]. However, the limited number of total articles on this topic, which has been acknowledged in multiple review articles in this cluster, suggests that more evidence is still needed to explore the association between gluten and MS [32,33].

As one of the most common symptoms in people with advanced neurological disability, dysphagia in MS formed a relatively small, and recent cluster (cluster 4). Studies in this cluster included the prevalence of dysphagia in MS [34,35], assessment of swallowing difficulties [36,37], and ways to improve the swallowing function [38]. Most of the studies were cross-sectional, short term or relatively small. Large cohort studies with a longitudinal design were still scant on this topic and need to be developed to better understand dysphagia in MS.

We identified gaps in the literature on diet and MS. Notably, disease progression, although mentioned in several articles, did not receive as much attention as the risk of MS. Few longitudinal studies were identified, which also showed that fewer studies have emphasised changes in the course of the disease. Further, a limited number of studies focused on the molecular mechanisms contributing to MS-specific pathological processes which highlighted that our understanding of biological mechanisms linking dietary factors to MS is still scant. Another gap was the lack of studies on specific foods or nutrients that could potentially decrease the risk of MS and/or attenuate disease progression.

The word cloud summarising the most investigated food items and nutrients showed that vitamin D takes a prominent place. Vitamin D was also the only nutrient that formed a single sub-cluster. Emerging from 1999, the number of articles published in each year in this sub-cluster peaked in 2010, during which 17 publications were found. The peak in 2010 was partially due to the review articles (9 out of 17 publications). After 2010, a decrease in articles numbers was seen until 2019, where a slight growth was detected again. In 2020, six articles were published in this cluster of vitamin D. Having been mentioned 131 times in all article titles, vitamin D is a relatively well researched nutrient. The most highly cited publication in this sub-cluster was a large prospective cohorts study addressing the protective effect of vitamin D on risk of MS published in 2004 [39]. The authors suggested that circulating levels of 25-hydroxyvitamin D (25(OH)D) should be measured, rather than estimating dietary intake of vitamin D. This might have influenced the later articles that cited this study [40].

Unlike vitamin D, articles mentioning “fat” or “fatty acid” in the title were fewer and did not form a separate sub-cluster. The earliest publications regarding fat or fatty acid dated back to 1991. Other nutrients appeared less frequently in titles. Between 1990 and 1999, articles regarding protein, cholesterol, vitamin B, tellurium, zinc, magnesium, and calcium emerged. Articles about sodium, iron, alcohol, and vitamin A, E, and C only appeared after 2000. Fibre, water, and caffeine were seen in titles only after 2010 in several articles. There were few articles about specific foods. The most frequently mentioned food item was salt, followed by olive oil, milk/dairy, and fish. The items investigated in the 1990s were fish, dairy, olive oil and garlic. Articles mentioning sugar and sweeteners, green tea, blueberry, egg, and salt then emerged. In the past five years, researchers started to study coffee, curcumin, pomegranate, meat, coconut oil, mushroom, and seaweed in relation to MS or other autoimmune and neurodegenerative diseases. The investigation of these new foods and their effects on other diseases could inspire studies on MS. Coffee, for example, was suggested to modulate the immune system because of its anti-inflammatory compound—caffeine [41]. A review showed that the studies on coffee and rheumatoid arthritis, psoriasis, and type 1 diabetes mellitus occurred earlier than that on MS [41]. Seaweed is another example that has shown therapeutic properties in Parkinson’s disease and Alzheimer’s disease because of its composition of oligosaccharides, long-chained polyunsaturated fatty acids, and bioactive amino acids and peptides which may have neuroprotective properties [42,43]. Therefore, more detailed investigations of the effects of seaweeds on MS in future studies may be warranted.

This data-driven literature map has several strengths. Chiefly, it provided a holistic view of the literature around diet and MS. Since the citations of publications were also included in the analysis, this data-driven literature map was able to cover a broader range of articles and reveal links that conventional reviews might overlook. Hierarchies between sub-clusters were also revealed by the literature map, which showed the subordinative relationship between topics. Studies were not separated by interventional or observational studies, but by topic, which allowed for the combination of evidence from human, animal and in vitro studies within clusters. Additionally, the combination of citation networks, word clouds and a literature map is a novel and intuitive way to present the trends and gaps in the literature. For researchers who want to compose systematic reviews or meta-analyses, it is a practical supplementary method for literature searching.

We identified some limitations relating to the search methodology. Firstly, we only used one literature source, the Web of Science Core Collection Database, thus only articles available in this database were included for analysis. This is due to a technical limitation of CitNetExplorer, which can only use the format exported from the Web of Science Core Collection Database. Additionally, influential studies on diet and MS may have been published before research was indexed in databases and therefore could not be considered in this analysis, such as the early articles of Roy Swank, who developed the Swank Diet for people with MS [44,45]. However, later studies based on the findings of those early articles from Roy Swank [46,47], as well as other studies about the Swank diet [48,49] were included in the current literature map. Further, keywords that appeared in abstracts or main bodies of articles, but not in titles, could have been overlooked. Additionally, a substantial proportion of publications were omitted as they did not form a cluster. Some of these articles had common topics but there were not enough articles to form a cluster. Other omitted articles had similar topics to those that formed clusters but did not have sufficient citation relationships. Nevertheless, there may be new clusters emerging when new topics receive more attention in the future. In addition, the data-driven review may not be able to highlight all gaps in the literature, especially small gaps in specific topics in sub-clusters; nevertheless, interested researchers can locate the specific topics of interest in the literature map and inspect their components in more detail to inform the direction of future research.

In summary, this literature map has revealed trends and gaps in the research on diet and MS. Most of the studies focused on MS risk and symptoms, rather than the longitudinal progression of the disease over a long time period. Many nutrients and foods that could potentially affect MS, such as antioxidants and foods with anti-inflammatory properties, still need further investigation. Further, it is important to investigate dietary patterns taking the whole diet and the effect of food combinations into account, exploring relationships between certain eating styles and MS onset and progression. Future studies could focus on filling these gaps, providing a stronger evidence base on the role of diet in MS.

## Data Availability

Not applicable.

## Supplementary Materials

The following are available online at https://www.mdpi.com/article/10.3390/nu14224820/s1, Table S1: Cluster 1: *n* = 728 publications, topic of diet and nutrition in MS risk and symptom management, refs [39,50,51,52,53,54,55,56,57,58,59,60,61], Table S2: Cluster 2: *n* = 166 publications, topic of experiments on cuprizone mouse models of MS, refs [19,27], Table S3: Cluster 3: *n* = 32 publications, topic of diet and gluten sensitivity in MS and other neurological diseases, ref [33], Table S4: Cluster 4: *n* = 22 publications, topic of dysphagia in MS and other neurologic diseases, and the influence of nutrition), ref [62]; and Supplementary File S1: All retrieved articles by clusters.
