# Supplementary material for: Mapping the Literature on Diet and Multiple Sclerosis: A Data-Driven Approach
Source: Nutrients. 2022 Nov 14;14(22):4820. doi: 10.3390/nu14224820 (PMC9696310; doi:10.3390/nu14224820)
Supplement: Supplementary file 1 [file nutrients-14-04820-s001.zip › Supplementary Tables 1-4.pdf]

Supplementary Table S1. Cluster 1: 728 publications, diet and nutrition in MS risk and symptom management

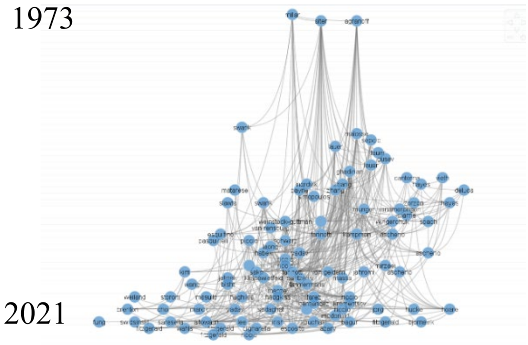

Citation network

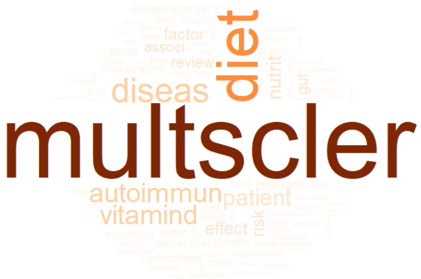

Cluster information and example publication	Word cloud	Most unique topic terms (relative frequency)
**1a: *n* = 369, diet and nutrition as risk factor for MS.**<br>Zhang SM, Hernán MA, Olek MJ, Spiegelman D, Willett WC, Ascherio A. Intakes of carotenoids, vitamin C, and vitamin E and MS risk among two large cohorts of women. Neurology. 2001 Jul 10;57(1):75-80. doi: 10.1212/wnl.57.1.75. PMID: 11445631 [1].	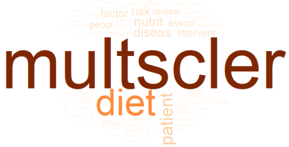	multscler* (8.57%)<br>diet* (4.40%)<br>patient (1.99%)<br>nutrit* (1.47%)<br>risk (1.30%)
**1aa: *n* = 217, associations between dietary factors and risk of MS.**<br>Pantzaris MC, Loukaides GN, Ntzani EE, Patrikios IS. A novel oral nutraceutical formula of omega-3 and omega-6 fatty acids with vitamins (PLP10) in relapsing remitting multiple sclerosis: a randomised, double-blind, placebo-controlled proof-of-concept clinical trial. BMJ Open. 2013 Apr 17;3(4):e002170. doi: 10.1136/bmjopen-2012-002170. PMID: 23599375; PMCID: PMC3641495 [2].	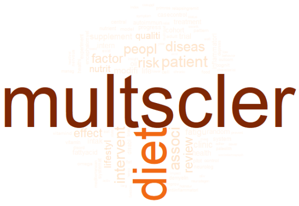	diet* (4.84%)<br>associ* (1.45%)<br>intervent* (1.40%)<br>risk (1.35%)<br>people* (1.30%)
**1aaa: *n* = 158, effect of dietary intervention among people with MS – clinical trials and reviews.**<br>Mauriz E, Laliena A, Vallejo D, Tuñón MJ, Rodríguez-López JM, Rodríguez-Pérez R, García-Fernández MC. Effects of a low-fat diet with antioxidant supplementation on biochemical markers of multiple sclerosis long-term care residents. Nutr Hosp. 2013 Nov 1;28(6):2229-35. PMID: 24506405 [3].	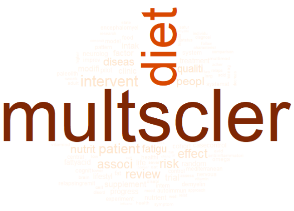	multscler* (7.90%)<br>diet (5.31%)<br>intervent* (1.54%)<br>associ* (1.26%)<br>people* (1.12%)
**1aab: *n* = 40, dietary compounds with anti-inflammatory properties and MS.**<br>Da Costa Silva BY, De Carvalho Sampaio HA, Shivappa N, Hébert J, Silva Albuquerque LD, Ferreira Carioca AA, Costa D'Almeida JA, Costa Maia CS, Pereira De Melo ML.	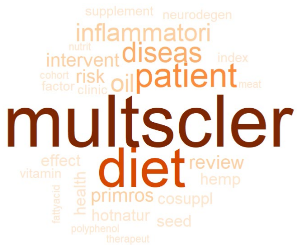	patient (2.79%)<br>diseas* (2.28%)<br>inflammatori* (2.03%)<br>oil (2.03%)<br>primros* (1.52%)<br>review (1.52%)<br>risk (1.52%)

Interactions between dietary inflammatory index, nutritional state and Multiple Sclerosis clinical condition. Clin Nutr ESPEN. 2018 Aug;26:35-41. doi: 10.1016/j.clnesp.2018.04.018. Epub 2018 May 18. PMID: 29908680 [4].		
**1ab: *n* = 142, special diets and nutrients used by people with MS (focusing on complementary and alternative medicine).** Yadav V, Shinto L, Bourdette D. Complementary and alternative medicine for the treatment of multiple sclerosis. Expert Rev Clin Immunol. 2010;6(3):381-395. doi:10.1586/eci.10.12 [5].	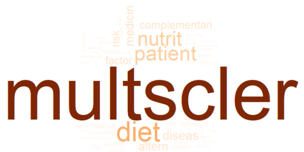	multscler* (10.67%)<br>patient (2.87%)<br>nutrit* (2.77%)<br>diseas* (1.64%)<br>altern* (1.54%)<br>medicin* (1.54%)
**1b: *n*=218, roles of diet and gut microbiota in autoimmune diseases.** Libbey JE, Sanchez JM, Doty DJ, Sim JT, Cusick MF, Cox JE, Fischer KF, Round JL, Fujinami RS. Variations in diet cause alterations in microbiota and metabolites that follow changes in disease severity in a multiple sclerosis model. Benef Microbes. 2018 Apr 25;9(3):495-513. doi: 10.3920/BM2017.0116. Epub 2018 Jan 30. PMID: 29380645; PMCID: PMC5918152 [6].	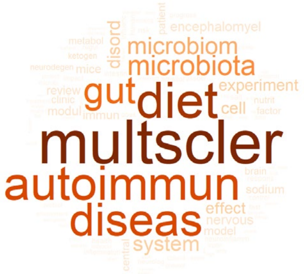	autoimmun* (3.41%)<br>diseas* (3.30%)<br>gut (2.66%)<br>microbiota (1.97%)<br>microbiom* (1.68%)
**1ba: *n* = 142, salt intake, gut microbiota and autoimmune disease.** Hucke S, Eschborn M, Liebmann M, Herold M, Freise N, Engbers A, Ehling P, Meuth SG, Roth J, Kuhlmann T, Wiendl H, Klotz L. Sodium chloride promotes pro-inflammatory macrophage polarization thereby aggravating CNS autoimmunity. J Autoimmun. 2016 Feb;67:90-101. doi: 10.1016/j.jaut.2015.11.001. Epub 2015 Nov 14. PMID: 26584738 [7].	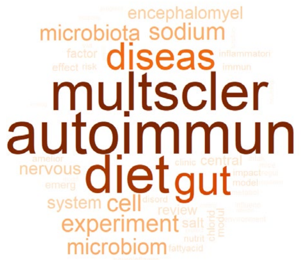	autoimmune* (4.92%)<br>diet* (4.38%)<br>cell (1.92%)<br>experiment (1.92%)<br>sodium (1.92%)
**1bb: *n* = 60, diets such as ketogenic diets and autoimmune diseases.** Bock M, Karber M, Kuhn H. Ketogenic diets attenuate cyclooxygenase and lipoxygenase gene expression in multiple sclerosis. EBioMedicine. 2018 Oct;36:293-303. doi: 10.1016/j.ebiom.2018.08.057. Epub 2018 Oct 3. PMID: 30292675; PMCID: PMC6197715 [8].	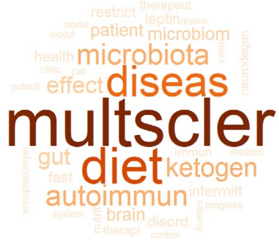	multscler* (5.22%)<br>ketogen* (1.96%)<br>effect (1.74%)<br>brain (1.30%)<br>patient (1.30%)
**1bc: *n* = 35, gut microbiota and autoimmune diseases - pathophysiology perspective.** Morshedi M, Hashemi R, Moazzen S, Sahebkar A, Hosseinifard ES. Immunomodulatory and anti-inflammatory effects of probiotics in multiple sclerosis: a systematic review. J Neuroinflammation. 2019 Nov 21;16(1):231. doi: 10.1186/s12974-019-1611-4. PMID: 31752913; PMCID: PMC6868771 [9].	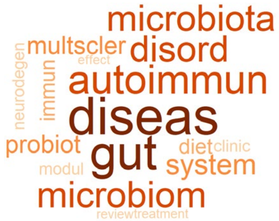	diseas* (4.98%)<br>gut (4.60%)<br>disord* (3.45%(<br>microbiom* (3.45%)<br>microbiota (3.45%)
**1bd: *n* = 30, obesity and central nervous dysfunction in mice models.** Langley MR, Yoon H, Kim HN, Choi CI, Simon W, Kleppe L, Lanza IR, LeBrasseur NK, Matveyenko A, Scarisbrick IA. High fat diet consumption results in mitochondrial dysfunction, oxidative stress,	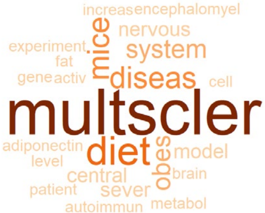	mice (2.17%)<br>obes* (1.81%)<br>central (1.44%)<br>model (1.44%)<br>nervous (1.44%)<br>sever (1.44%)

and oligodendrocyte loss in the central nervous system. Biochim Biophys Acta Mol Basis Dis. 2020 Mar 1;1866(3):165630. doi: 10.1016/j.bbadis.2019.165630. Epub 2019 Dec 6. PMID: 31816440; PMCID: PMC7982965 [10].		
**1c: *n* = 104, effect of vitamin D in MS - human and mice studies.** Wingerchuk DM, Lesaux J, Rice GPA, et alA pilot study of oral calcitriol (1,25-dihydroxyvitamin D3) for relapsing–remitting multiple sclerosisJournal of Neurology, Neurosurgery & Psychiatry 2005;76:1294-1296 [11].	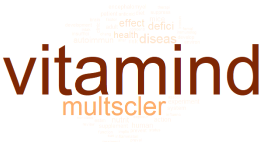	vitamind* (10.99%)<br>defici* (1.51%)<br>effect (1.37%)<br>health (1.24%)<br>human (0.96%)<br>mice (0.96%)
**1ca: *n* = 45, vitamin D deficiency and risk of developing MS.** Munger KL, Zhang SM, O'Reilly E, Hernán MA, Olek MJ, Willett WC, Ascherio A. Vitamin D intake and incidence of multiple sclerosis. Neurology. 2004 Jan 13;62(1):60-5. doi: 10.1212/01.wnl.0000101723.79681.38. PMID: 14718698 [12].	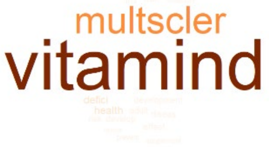	multscler* (5.99%)<br>defici* (1.50%)<br>health (1.50%)<br>adult (1.20%)<br>develop* (1.20%)<br>development (1.20%)<br>risk (1.20%)
**1cb: *n* = 30, effect of vitamin D on MS, especially in mice models.** DeLuca HF, Plum LA. Vitamin D deficiency diminishes the severity and delays onset of experimental autoimmune encephalomyelitis. Arch Biochem Biophys. 2011;513(2):140-143. doi:10.1016/j.abb.2011.07.005 [13].	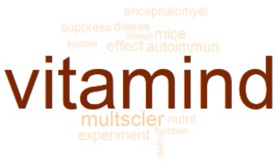	vitamind* (12.56%)<br>autoimmune* (2.42%)<br>effect (2.42%)<br>experiment (2.42%)<br>mice (2.42%)

Note: Citation network was a schematic overview of the cluster. Only top 100 most cited articles were shown. Colored nodes indicated core citations, lines between nodes showed citation links. Year range indicated the period covered by the publication in the cluster. Word clouds were based on raw word frequency, with larger size and opacity indicating higher frequency. Unique words were relative frequencies of words among same level sub-clusters. * Indicated word stem wildcard. Counts of articles added up in sub-clusters might exceed total cluster count, as single publications could belong to multiple clusters.

Supplementary Table S2. Cluster 2: 166 publications, experiments on cuprizone mouse models of MS

1996

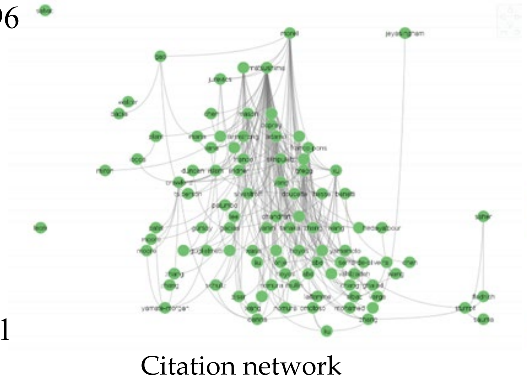

2021

Citation network

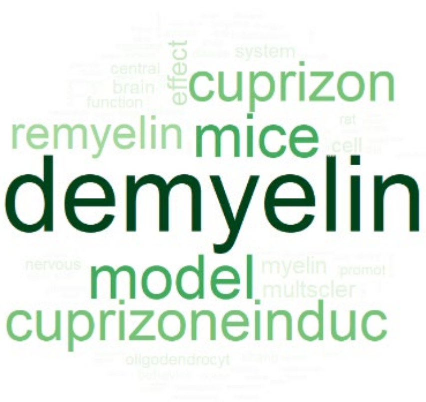

Cluster information and example publication	Word cloud	Most unique topic terms (relative frequency)

**2a: *n* = 121, de- and remyelination in cuprizone mouse models**<br>Liebetanz D, Merkler D. Effects of commissural de- and remyelination on motor skill behaviour in the cuprizone mouse model of multiple sclerosis. Exp Neurol. 2006 Nov;202(1):217-24. doi: 10.1016/j.expneurol.2006.05.032. Epub 2006 Jul 20. PMID: 16857191 [14].	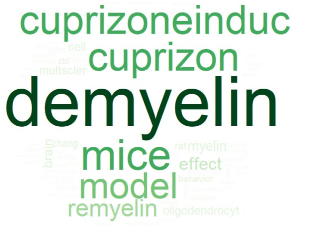	demyelin* (6.77%)<br>mice (4.15%)<br>cuprizon* (3.89%)<br>cuprizoneinduc* (3.89%)<br>myelin* (1.27%)
**2b: *n* = 20, promoting remyelination in cuprizone mouse models.**<br>Zhang J, Zhang ZG, Lu M, Wang X, Shang X, Elias SB, Chopp M. MiR-146a promotes remyelination in a cuprizone model of demyelinating injury. Neuroscience. 2017 Apr 21;348:252-263. doi: 10.1016/j.neuroscience.2017.02.029. Epub 2017 Feb 23. PMID: 28237816 [15].	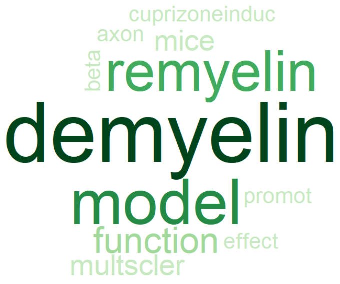	model (4.89%)<br>remyelin* (4.35%)<br>function (2.72%)<br>multscler* (2.17%)<br>axon (1.63%)<br>beta (1.63%)<br>effect (1.63%)<br>promot* (1.63%)

Note: Citation network was a schematic overview of the cluster. Only top 100 most cited articles were shown. Colored nodes indicated core citations, lines between nodes showed citation links. Year range indicated the period covered by the publication in the cluster. Word clouds were based on raw word frequency, with larger size and opacity indicating higher frequency. Unique words were relative frequencies of words among same level sub-clusters. * Indicated word stem wildcard. Counts of articles added up in sub-clusters might exceed total cluster count, as single publications could belong to multiple clusters.

Supplementary Table S3. Cluster 3: 32 publications, diet and gluten sensitivity in MS and other neurological diseases

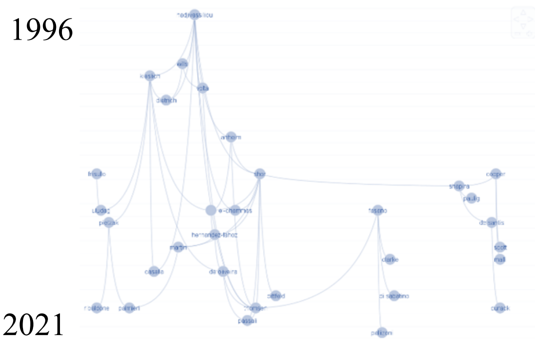

Citation network

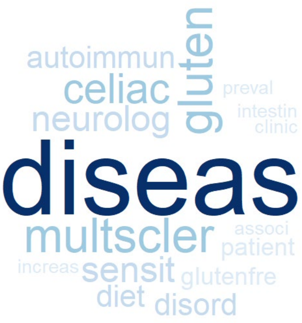

**Example publication**	**Most unique topic terms (relative frequency)**
Passali M, Josefsen K, Frederiksen JL, Antvorskov JC. Current Evidence on the Efficacy of Gluten-Free Diets in Multiple Sclerosis, Psoriasis, Type 1 Diabetes and Autoimmune Thyroid Diseases. Nutrients. 2020 Aug 1;12(8):2316. doi: 10.3390/nu12082316. PMID: 32752175; PMCID: PMC7468712 [16].	diseas* (8.53%)<br>gluten (3.79%)<br>celiac (3.32%)<br>sensit* (2.84%)<br>autoimmune* (2.37%)<br>disord* (2.37%)

Note: Citation network was a schematic overview of the cluster. Only top 100 most cited articles were shown. Colored nodes indicated core citations, lines between nodes showed citation links. Year range indicated the period covered by the publication in the cluster. Word clouds were based on raw word frequency, with larger size and opacity indicating higher frequency. Unique words were relative frequencies of words among same level sub-clusters. * Indicated word stem wildcard. Counts of articles added up in sub-clusters might exceed total cluster count, as single publications could belong to multiple clusters.

Supplementary Table S4. Cluster 4: 22 publications, dysphagia in MS and other neurologic diseases, and the influence of nutrition

1999

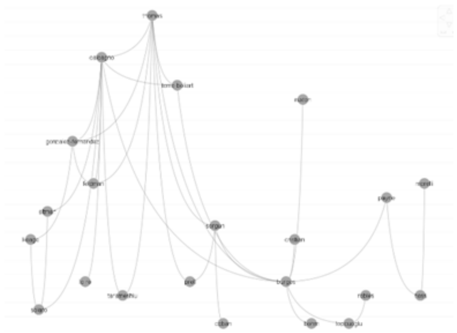

2021

Citation network

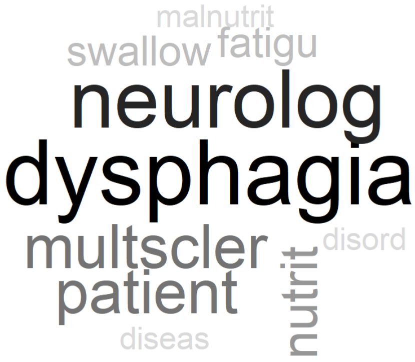

**Example publication**	**Most unique topic terms (relative frequency)**
Thomas FJ, Wiles CM. Dysphagia and nutritional status in multiple sclerosis. J Neurol. 1999 Aug;246(8):677-82. doi: 10.1007/s004150050431. PMID: 10460444 [17].	dysphagia (6.12%)<br>neurolog* (5.44%)<br>patient (4.08%)<br>nutrit* (3.40%)<br>fatigu* (2.72%)<br>swallow (2.72%)

Note: Citation network was a schematic overview of the cluster. Only top 100 most cited articles were shown. Colored nodes indicated core citations, lines between nodes showed citation links. Year range indicated the period covered by the publication in the cluster. Word clouds were based on raw word frequency, with larger size and opacity indicating higher frequency. Unique words were relative frequencies of words among same level sub-clusters. * Indicated word stem wildcard. Counts of articles added up in sub-clusters might exceed total cluster count, as single publications could belong to multiple clusters.

References

1. Zhang SM, Hernán MA, Olek MJ, Spiegelman D, Willett WC, Ascherio A. Intakes of carotenoids, vitamin C, and vitamin E and MS risk among two large cohorts of women. Neurology. 2001 Jul 10;57(1):75-80. doi: 10.1212/wnl.57.1.75. PMID: 11445631
2. Pantzaris MC, Loukaides GN, Ntzani EE, Patrikios IS. A novel oral nutraceutical formula of omega-3 and omega-6 fatty acids with vitamins (PLP10) in relapsing remitting multiple sclerosis: a randomised, double-blind, placebo-controlled proof-of-concept clinical trial. BMJ Open. 2013 Apr 17;3(4):e002170. doi: 10.1136/bmjopen-2012-002170. PMID: 23599375; PMCID: PMC3641495
3. Mauriz E, Laliena A, Vallejo D, Tuñón MJ, Rodríguez-López JM, Rodríguez-Pérez R, García-Fernández MC. Effects of a low-fat diet with antioxidant supplementation on biochemical markers of multiple sclerosis long-term care residents. Nutr Hosp. 2013 Nov 1;28(6):2229-35. PMID: 24506405
4. Da Costa Silva BY, De Carvalho Sampaio HA, Shivappa N, Hébert J, Silva Albuquerque LD, Ferreira Carioca AA, Costa D'Almeida JA, Costa Maia CS, Pereira De Melo ML. Interactions between dietary inflammatory index, nutritional state and Multiple Sclerosis clinical condition. Clin Nutr ESPEN. 2018 Aug;26:35-41. doi: 10.1016/j.clnesp.2018.04.018. Epub 2018 May 18. PMID: 29908680.
5. Yadav V, Shinto L, Bourdette D. Complementary and alternative medicine for the treatment of multiple sclerosis. Expert Rev Clin Immunol. 2010;6(3):381-395. doi:10.1586/eci.10.12
6. Libbey JE, Sanchez JM, Doty DJ, Sim JT, Cusick MF, Cox JE, Fischer KF, Round JL, Fujinami RS. Variations in diet cause alterations in microbiota and metabolites that follow changes in disease severity in a multiple sclerosis model. Benef Microbes. 2018 Apr 25;9(3):495-513. doi: 10.3920/BM2017.0116. Epub 2018 Jan 30. PMID: 29380645; PMCID: PMC5918152
7. Hucke S, Eschborn M, Liebmann M, Herold M, Freise N, Engbers A, Ehling P, Meuth SG, Roth J, Kuhlmann T, Wiendl H, Klotz L. Sodium chloride promotes pro-inflammatory macrophage polarization thereby

aggravating CNS autoimmunity. J Autoimmun. 2016 Feb;67:90-101. doi: 10.1016/j.jaut.2015.11.001. Epub 2015 Nov 14. PMID: 26584738

8. Bock M, Karber M, Kuhn H. Ketogenic diets attenuate cyclooxygenase and lipoxygenase gene expression in multiple sclerosis. EBioMedicine. 2018 Oct;36:293-303. doi: 10.1016/j.ebiom.2018.08.057. Epub 2018 Oct 3. PMID: 30292675; PMCID: PMC6197715
9. Morshedi M, Hashemi R, Moazzen S, Sahebkar A, Hosseinifard ES. Immunomodulatory and anti-inflammatory effects of probiotics in multiple sclerosis: a systematic review. J Neuroinflammation. 2019 Nov 21;16(1):231. doi: 10.1186/s12974-019-1611-4. PMID: 31752913; PMCID: PMC6868771
10. Langley MR, Yoon H, Kim HN, Choi CI, Simon W, Kleppe L, Lanza IR, LeBrasseur NK, Matveyenko A, Scarisbrick IA. High fat diet consumption results in mitochondrial dysfunction, oxidative stress, and oligodendrocyte loss in the central nervous system. Biochim Biophys Acta Mol Basis Dis. 2020 Mar 1;1866(3):165630. doi: 10.1016/j.bbadis.2019.165630. Epub 2019 Dec 6. PMID: 31816440; PMCID: PMC7982965
11. Wingerchuk DM, Lesaux J, Rice GPA, et alA pilot study of oral calcitriol (1,25-dihydroxyvitamin D3) for relapsing–remitting multiple sclerosisJournal of Neurology, Neurosurgery & Psychiatry 2005;76:1294-1296
12. Munger KL, Zhang SM, O'Reilly E, Hernán MA, Olek MJ, Willett WC, Ascherio A. Vitamin D intake and incidence of multiple sclerosis. Neurology. 2004 Jan 13;62(1):60-5. doi: 10.1212/01.wnl.0000101723.79681.38. PMID: 14718698
13. DeLuca HF, Plum LA. Vitamin D deficiency diminishes the severity and delays onset of experimental autoimmune encephalomyelitis. Arch Biochem Biophys. 2011;513(2):140-143. doi:10.1016/j.abb.2011.07.005.
14. Liebetanz D, Merkler D. Effects of commissural de- and remyelination on motor skill behaviour in the cuprizone mouse model of multiple sclerosis. Exp Neurol. 2006 Nov;202(1):217-24. doi: 10.1016/j.expneurol.2006.05.032. Epub 2006 Jul 20. PMID: 16857191
15. Zhang J, Zhang ZG, Lu M, Wang X, Shang X, Elias SB, Chopp M. MiR-146a promotes remyelination in a cuprizone model of demyelinating injury. Neuroscience. 2017 Apr 21;348:252-263. doi: 10.1016/j.neuroscience.2017.02.029. Epub 2017 Feb 23. PMID: 28237816
16. Passali M, Josefsen K, Frederiksen JL, Antvorskov JC. Current Evidence on the Efficacy of Gluten-Free Diets in Multiple Sclerosis, Psoriasis, Type 1 Diabetes and Autoimmune Thyroid Diseases. Nutrients. 2020 Aug 1;12(8):2316. doi: 10.3390/nu12082316. PMID: 32752175; PMCID: PMC7468712
17. Thomas FJ, Wiles CM. Dysphagia and nutritional status in multiple sclerosis. J Neurol. 1999 Aug;246(8):677-82. doi: 10.1007/s004150050431. PMID: 10460444.
